# Long Non-Coding RNA Expression Profile in the Kidneys of Male, Low Birth Weight Rats Exposed to Maternal Protein Restriction at Postnatal Day 1 and Day 10

**DOI:** 10.1371/journal.pone.0121587

**Published:** 2015-03-31

**Authors:** Yanhong Li, Xueqin Wang, Mengxia Li, Jian Pan, Meifang Jin, Jian Wang, Xiaozhong Li, Xing Feng

**Affiliations:** 1 Department of Nephrology, Children’s Hospital of Soochow University, Suzhou, China; 2 Institute of Pediatric Research, Children’s Hospital of Soochow University, Suzhou, China; 3 Department of Neonatology, Children’s Hospital of Soochow University, Suzhou, China; National Centre for Scientific Research “Demokritos”, GREECE

## Abstract

**Background:**

Long non-coding RNAs (lncRNAs), which are involved in a variety of biological functions and aberrantly expressed in many types of diseases, are required for postnatal development. In this study, we aimed to investigate the lncRNA profiles in low birth weight (LBW) rats with reduced nephron endowment induced by the restriction of maternal protein intake. LBW by reduced nephron endowment is a risk factor for hypertension and end-stage renal disease in adulthood.

**Methods:**

Kidneys were obtained from LBW rats fed a low-protein diet throughout gestation and lactation as well as from normal control rats born from dams fed normal protein diets at postnatal day 1 (p1) and 10 (p10). The total number of glomeruli in the kidneys was counted at p10. LncRNA expression profiles were analyzed by sequencing and screening using the Agilent Rat lncRNA Array. Quantitative real-time PCR (qRT-PCR) analysis of these lncRNAs confirmed the identity of some genes.

**Results:**

The total number of glomeruli per kidney at p10 was significantly lower in LBW rats than in controls. A total of 42 lncRNAs were identified to be significantly differentially expressed, with fold-changes ≥2.0, between the two groups. According to correlation analysis between the differentially expressed lncRNAs and mRNAs involved in kidney development, we randomly selected a number of lncRNAs for comparison analysis between LBW and control kidneys at the two time-points, p1 and p10, using qRT-PCR. Three lncRNAs (TCONS_00014139, TCONS_00014138, and TCONS_00017119), which were significantly correlated with the mRNA expression of mitogen-activated protein kinase 4, were aberrantly expressed in LBW rats, compared with controls, at both p1 and p10.

**Conclusions:**

LncRNAs are aberrantly expressed in the kidneys of LBW rats, compared with controls, during nephron development, which indicates that lncRNAs might be involved in impaired nephron endowment.

## Introduction

Low birth weight (LBW) induced by intrauterine growth restriction (IUGR) is considered to be a predisposing factor for hypertension and renal disease in adulthood [1–3]. IUGR often leads to reduced nephron endowment in LBW infants. A linear relationship between nephron number and birth weight was previously identified in children and adults [4]. Reduced nephron endowment at the beginning of life may be subsequently cause a long-term risk of hypertension and renal disease in adult life [5–8]. However, the underlying mechanism of how LBW is linked to reduced nephron endowment remains to be established.

Long non-coding RNAs (lncRNAs) are defined as non-coding RNAs that are longer than 200 nucleotides in length [9]. Accumulated evidence has indicated that lncRNAs exhibit important roles in various biological processes [9, 10]. The aberrant regulation of lncRNAs has been shown to be associated with a variety of human diseases, such as neurological disorders, heart diseases, and kidney disorders [11–14].

To date, a few studies on the roles of lncRNAs, as crucial regulators, during normal development have been reported [15–19]. Sauvageau et al. revealed that lncRNAs are required for brain development by using multiple knockout mouse models [17]. Zhu et al. suggested that lncRNAs might be involved in heart development [18]. In renal development, a previous study suggested that mesodermal specific cDNA or transcripts and H19, an imprinted gene, are developmentally regulated, and their concomitant decreased expression might be responsible for the perturbed epithelial and mesenchymal interactions leading to dysmorphogenesis of the metanephros [20]. However, little is known about the overall expression status of lncRNAs during nephron development. The purpose of the study is to investigate the lncRNA profiles in LBW rat kidneys with low nephron number induced by the restriction of maternal protein intake, compared to normal controls. This would enable us to understand whether lncRNAs might play a role in reduced nephron endowment.

## Materials and Methods

### Ethics Statement

This study was conducted in strict accordance with the recommendations in the Guide for the Care and Use of Laboratory Animals of the National Institutes of Health. The protocol was approved by the Committee on the Ethics of Animal Experiments of the Children’s Hospital of Soochow University. All surgery was performed under 10% chloral hydrate anesthesia, and all efforts were made to minimize suffering of rats.

### Animals

Sprague-Dawley rats, weighing approximately 200–250 g, were obtained from the JOINN Laboratories, Inc., SuZhou, China (Grade II), and bred in the *animal laboratory* of Soochow University Medical Center for two weeks before mating. The female rats were mated by exposure to males. Pregnant rats, which were confirmed by the presence of sperm in the vaginal smear, were randomly fed either a normal protein diet (22.2% protein) or an isocaloric low-protein diet (6.6% protein) to induce LBW during the pregnancy. Food was available *ad libitum*. The composition of these diets is shown in Table 1. During the experiment period, the animals were maintained with a 12-h light/dark cycle, in climate controlled and pathogen-free conditions. The rats had free access to tap water. All pups stayed with their dams until sacrificed. The dams were maintained on normal or low-protein diets during the first 10 days of the lactation period. Litters with larger (>16) and smaller (<6) pups were considered abnormal and were excluded from the study to ensure an adequate and standardized nutrient supply. The number of pups per litter was similar between the two groups.

**Table 1:** Composition of normal and low protein diets fed to dams during pregnancy and the first 10 days of lactation period.

	Composition (% by weight)	Protein (%)	Energy (KJ/100g)
Normal protein diet	Wheat 34%, maize 26%, soybean powder 27%, fishmeal 5%, alfalfa powder 2%, rapeseed oil 1%, premix 5%	22.2	1620.4
Low-protein diet	Maize 50%, wheat 26.5%, sucrose 13%, fishmeal 3%, rapeseed oil 2.5%, premix 5%	6.6	1568.7

Birth weights were measured on the first day of postnatal life within 12 h after delivery. LBW was defined as a birth weight <2 *standard deviation* (SD) below the mean of the control pups (mean-2SD) that were born from dams fed normal protein diets. Because nephrogenesis continues after birth until postnatal days 7–10 in rats [21, 22], LBW rats born from dams fed low-protein diets and normal birth weight controls from dams fed norm protein diets were investigated on both postnatal day 1 (p1) and day 10 (p10) of life. Seven rats were randomly selected from each group at each time point of the study. Because there was a significant difference between male and female LBW rats in the total number of glomeruli per kidney at p10 and creatinine clearance in adulthood (data not shown), only newborn male rats were used in the study to avoid possible gender differences.

### Collection of kidney tissues

LBW and normal control rats at p1 or p10 were anesthetized with 10% chloral hydrate (300–500 mg/kg), which was given intraperitoneally after weighing. Both kidneys were removed and weighed. The right kidney was used for the immediate estimation of glomerular number. The left kidney was rapidly harvested, rinsed in saline, and kept frozen at -80°C until the measurements of tissue lncRNA and mRNA levels.

To assess the expression of lncRNAs in different tissues, LBW and normal control male rats at p10 were anesthetized, and their kidney, liver, brain, and lung were excised for the measurement of lncRNAs by quantitative real-time PCR (qRT-PCR) in a separate experiment.

### Glomerular count

The total number of glomeruli was determined using the modified maceration method [23, 24]. Briefly, the right kidney from LBW and control rats at p10 was cut into small pieces and incubated in 1% ammonium chloride for 2 h at room temperature, followed by gentle pipetting in 20 mL of 50% hydrochloric acid for 90 min at 37°C. The tissue was homogenized through repeated pipetting, and incubated in 45 mL phosphate buffered saline overnight at 4°C. After slow-speed centrifugation, the resultant pellet, containing the glomeruli, was resuspended in 10 mL distilled water. Twenty 20-μL aliquots were pipetted onto counting slides and covered with slips. The number of glomeruli in the aliquot was counted at 10 x magnification. The total number of glomeruli per kidney was extrapolated mathematically from the counting of all the aliquots.

### Microarray expression analysis

The Agilent Rat lncRNA Array was designed for profiling both lncRNAs and protein coding RNAs of the rat genome (8*60 K, Design ID: 062716). A total of 9,984 lncRNAs and 30,367 coding transcripts were collected from the most authoritative databases, including RefSeq, UCSC Knowngenes, Ensembl, and other related literature. Three rats at p10 were randomly selected from each group for the microarray expression analysis.

The microarray analysis was performed based on the manufacturer’s standard protocols. Briefly, RNA derived from the rat kidneys was extracted using the mirVana RNA Isolation Kit. RNA quality and quantity were evaluated using a NanoDrop ND-2000 Spectrophotometer and RNA integrity was assessed by Agilent Bioanalyzer 2100. After assessment, total RNA were transcribed to double-stranded cDNA, then synthesized into fluorescent cRNA and labeled with Cyanine-3-CTP using the Quick Amp labeling kit. According to the Agilent One-Color Microarray-Based Gene Expression Analysis protocol, the labeled cRNAs were hybridized onto the microarray. After washing, the hybridized arrays were fixed and scanned using an Agilent DNA microarray scanner (G2505C). Raw and processed microarray data have been deposited in the National Center for Biotechnology Information (NCBI) Gene Expression Omnibus (GEO) and are accessible through (GEO) series accession number, GSE64239.

### Microarray data analysis

Agilent Feature Extraction software (version 10.7.1.1) was used to analyze array images. Quantile normalization and subsequent data processing were performed using Agilent GeneSpring GX software (version 12.5). Differentially expressed lncRNAs and mRNAs were identified through fold change and *p* values. The threshold used to screen up/down-regulated genes was a fold change ≥2.0 and a *p* value ≤0.05, as calculated with Student’s t-test. Hierarchical clustering was performed to display the distinguishable expression pattern of the lncRNAs among samples.

### Bioinformatics analysis

Because lncRNAs have been shown to be preferentially located next to genes with developmental functions [9, 25], the nearest protein-coding gene was identified for each lncRNA locus using UCSC Genome Bioinformatic tools (http://genome.ucsc.edu). In addition, correlation analysis between differentially expressed lncRNAs and mRNAs from microarray data was performed to investigate the relationship between lncRNAs and their coding genes. Pearson correlation coefficients ≥0.90 with *p* value <0.05 indicate a significant relationship.

### Quantitative real-time PCR

Total RNA was extracted from kidney, liver, brain, and lung tissues obtained from LBW and normal control rats at p1 or p10 using the mirVana RNA Isolation Kit. For reverse transcription-PCR, cDNA was synthesized in a 10 μl reaction volume containing 0.5 μg RNA, 2 μl of PrimerScript buffer, 0.5 μl of oligo dT, 0.5 μl of random 6mers, and 0.5 μl of PrimerScript RT Enzyme Mix I. The reactions were conducted in a GeneAmp PCR System 9700 for 15 min at 37°C, followed by heat inactivation of reverse transcription for 5 s at 85°C, according to the manufacturer’s instructions.

Quantitative RT-PCR was performed on the LightCycler 480 Ⅱ Real-time PCR Instrument with 1 μl reverse transcription product, 0.2 μl forward and reverse primers, and 5 μl of 2 × LightCycler 480 SYBR Green I Master mix. The PCR conditions included an initial denaturation step of 10 min at 95°C, followed by 40 cycles of amplification and quantification (95°C for 10 s and 60°C for 30 s). Each sample was measured in triplicate. The specificity of the expected PCR product was ensured by melting curve analysis and electrophoresis on agarose gels (data not shown). The gene expression of all the target sequences was normalized to the expression of an endogenous control, glyceraldehyde 3-phosphate dehydrogenase (GAPDH). The mean of each triplicate was used to calculate the relative concentration of the lncRNAs (Ct = Ct mean lncRNAs-Ct mean GAPDH). The fold changes in lncRNA expression between LBW and control rats were calculated by the 2^-ΔΔCt^ method. The sequences of the primers, which were designed in the laboratory and synthesized by Generay Biotech, are listed in Table 2.

**Table 2:** Real-Time PCR Primers for 5 lncRNAs and GAPDH.

Gene	Forward primer	Reverse primer
TCONS_00116697	GTGGGCATCCTGTTCATATC	CTAGCAGGACCTCATTCATTT
TCONS_00038464	TCCCTTCTCCCTGCTTTC	CCCACAATCATGTTTGACCA
TCONS_00014139	CACCACCAGTTATAAGCG	TGACGGATGAGCCGATAC
TCONS_00014138	AGTATCCAGTAAGCGGTG	TCTGGCTTCCTGAGCATAG
TCONS_00017119	CAGCAGATGAAGTGATATCTCG	CAGAAGAGGGCATTAAATTACC
GAPDH	GCGAGATCCCGCTAACATCA	CTCGTGGTTCACACCCATCA

GAPDH, glyceraldehyde 3-phosphate dehydrogenase

### Statistical analysis

Statistical analyses were performed using SPSS Statistics 13.0. Results are presented as mean±SD. Differences between LBW and control rats were analyzed using the Student’s t-test or the Mann-Whitney U-test. Spearman correlation was used to examine the relationship between glomerular number and body weight at birth. A value of *p* <0.05 was considered to be statistically significant.

## Results

### Body weight and glomerular number in the LBW rats compared with the normal control rats

The exposure to a low-protein diet throughout gestation induced IUGR in rats. There was a significant difference in the birth weight of pups of dams fed with normal and low-protein diets. All pups born from dams fed low-protein diets developed LBW, defined as a birth weight <6.0 g (mean-2SD of the control pups born from dams fed normal protein diets).

Male rats with LBW born from dams fed a low-protein diet throughout gestation and the first 10 days of lactation period and control male rats with normal birth weight born from dams fed normal protein diets were randomly selected for the study (n = 7 for each group at each time point). The total number of glomeruli in the right kidney was counted at p10 of life.

**Fig 1:**
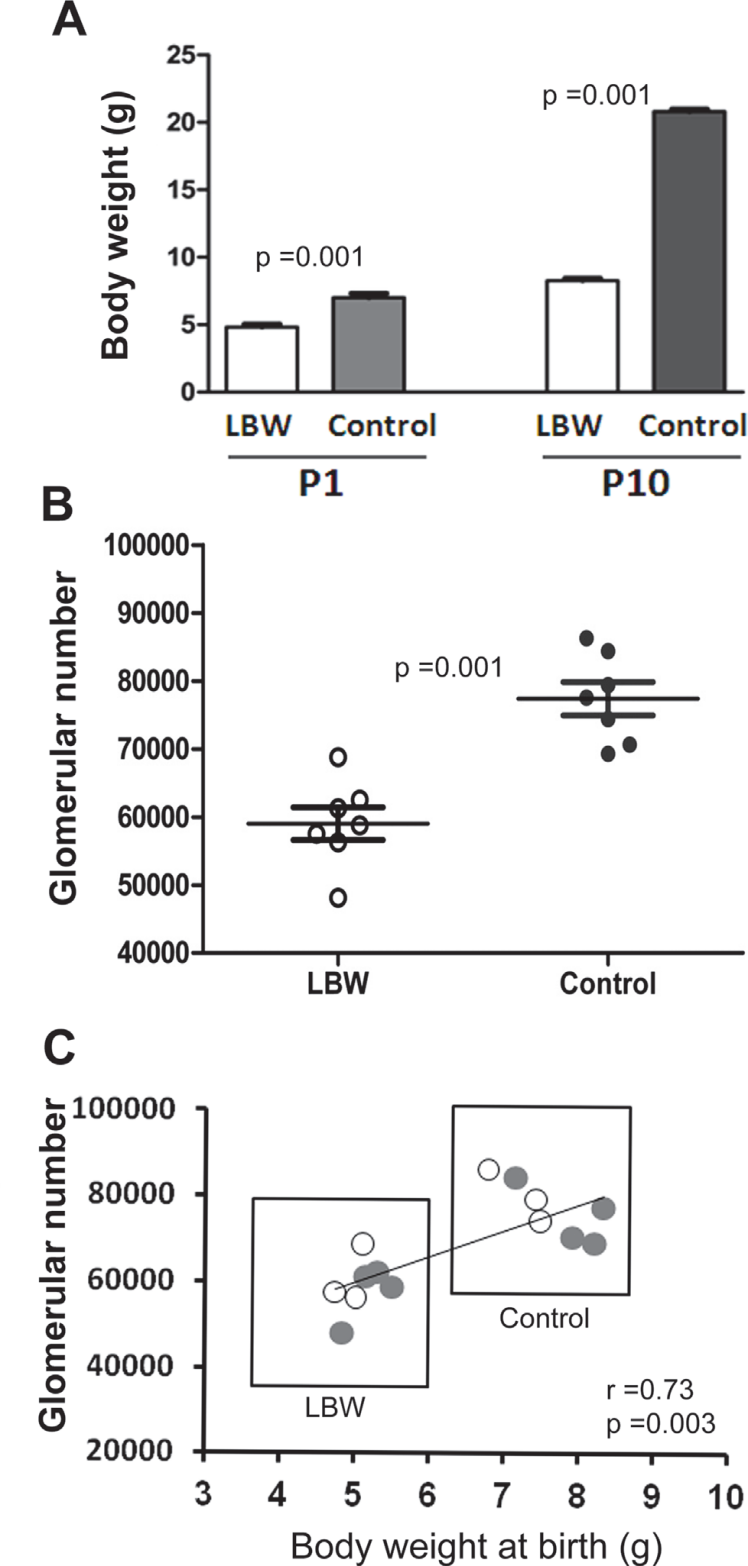
Body weight and glomerular number in low birth weight rats compared with controls. **A:** Body weight in low birth weight (LBW) rats compared with normal controls at postnatal day 1 (p1) and 10 (p10). Values are means with SD. **B:** Comparison of glomerular number between LBW and control rats at p10. Each circle represents an individual rat and the horizontal and vertical lines indicate the means and SD. *p* Value between LBW and control rats was calculated by the Mann-Whitney U-test, n *=* 7 for each group. **C:** Scatter plot showing the distribution of glomerular number based on birth weight. The number of glomeruli was positively correlated with body weight at birth. r = Spearman’s correlation coefficient. The open circles represent the three rats randomly selected for microarray analysis from each group.

As shown in Fig 1A, LBW rats had a significantly lower body weight compared with controls at p1 (4.8 ± 0.6 vs. 7.0 ± 0.8 g; *p* = 0.001; n = 7). The difference in body weight remained statistically significant at p10 (8.3 ± 0.5 vs. 20.8 ± 0.5 g; *p* = 0.001; n = 7). The total number of glomeruli per kidney at p10 was also significantly lower in LBW rats than in control rats (59017 ± 6331 vs. 77392 ± 6485; *p* = 0.001; n = 7).The glomerular number and the distribution of glomerular number by birth weight are shown in Fig 1B and 1C.

### LncRNAs are aberrantly expressed in the kidneys of LBW rats compared with the normal controls

**Fig 2:**
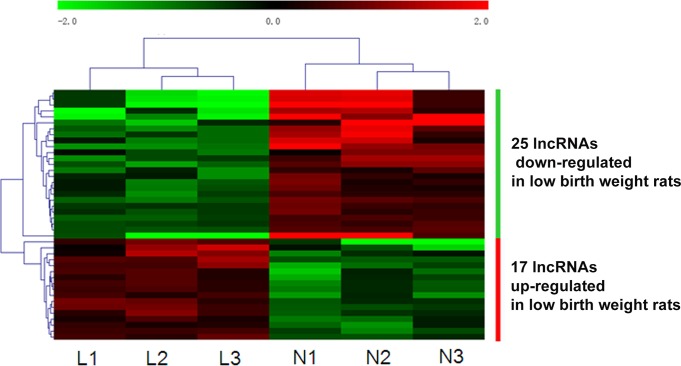
Hierarchical clustering of lncRNAs differentially expressed in kidney between low birth weight and control rates. A hierarchical clustered heat map showing the log_2_ transformed expression values for differentially expressed lncRNAs (absolute fold-change ≥2; *p* ≤0.05) between low birth weight rats (L) and normal controls (C). Three rats were analyzed for each group. The intensity of the color scheme is calibrated to the log_2_ expression values, where red refers to high relative expression and green refers to low relative expression. The bar code represents the color scale of the log_2_ values.

To study the potential role of lncRNAs in the development of nephrons, we used a second-generation LncRNA microarray to determine the lncRNA and mRNA expression profiles in the kidneys of p10 rats with low glomerular number. Three rats were randomly selected from each group at p10 (indicated by open circles in Fig 1C). In total, 8,941 lncRNAs and 27,697 coding transcripts were detected by the microarray. As shown in S1 and S2 Tables, the expression patterns of lncRNA and mRNA in the kidneys between LBW and control rats were significantly different. A total of 42 lncRNAs were identified to be differentially expressed with more than 2-fold change (Table 3). Among them, 17 and 25 were up and down expressed in LBW rats compared with normal controls, respectively. The most down-regulated lncRNAs in the kidneys with low nephron number were TCONS_00098342, TCONS_00075112, TCONS_00106261, TCONS_00031872 TCONS_00139993 TCONS_00134056, TCONS_00025089, and TCONS_00116697 (absolute fold-change ≥5), and the most up-regulated lncRNAs were TCONS_00074456, TCONS_00036328, and TCONS_00017119 (absolute fold-change ≥3), as shown in Table 4. The nearest protein-coding gene identified for these lncRNAs using UCSC Genome Browser at a distance of <10 kb is also shown in Table 4. Hierarchical clustering of the differentially expressed lncRNAs is shown in Fig 2 (n = 3 for each group).

**Table 3:** Relative differential lncRNA and mRNA expression in the kidneys between low birth weight and control rats at postnatal day 10 of life, detected using microarray.

	Fold change ≥2	Fold change ≥4	Fold change ≥6	total
Long non-coding RNA				
up-regulated	16	0	1	17
down-regulated	16	4	5	25
Message RNA				
up-regulated	38	2	1	41
down-regulated	74	7	7	88

**Table 4:** The most down/up-regulated lncRNAs in the kidneys of low birth weight rats compared with normal controls (microarray data).

LncRNA	Regulation	Fold change^a^	Chromosomal localization	Start locus	Stop locus	Associated gene name^b^
TCONS_00098342	down	22.628	4	165983643	165984534	Clec2d
TCONS_00075112	down	11.205	2	158038578	158039601	NA
TCONS_00106261	down	8.905	5	746953	747160	NA
TCONS_00031872	down	6.576	11	56851598	56852246	Phactr4
TCONS_00139993	down	6.351	X	71738347	71738923	Dmd
TCONS_00134056	down	5.669	9	63737838	63744419	Crygb
TCONS_00025089	down	5.473	10	71300611	71301180	Slfn3
TCONS_00116697	down	5.306	7	127195009	127218521	Pim3
TCONS_00074456	up	10.937	2	39476293	39476907	Ercc8
TCONS_00036328	up	3.395	12	35676307	35677577	Myl2
TCONS_00017119	up	3.165	1	215808322	215808977	Keg1

Clec2d, C-type lectin domain family 2, member D; Crygb, crystalline, gamma B; Dmd, dystrophin; Ercc8, excision repair cross-complementation group 8; Keg1, glycine-N-acyltransferase-like 2 (Glyatl2); Myl2, myosin, light chain 2; Phactr4, phosphatase and actin regulator 4; Pim3, Pim-3 proto-oncogene, serine/threonine kinase; Slfn3, schlafen 3; NA, not applicable.

^a^Values indicate the absolute fold-change between low birth weight rats compared with normal controls detected by microarray.

^b^Nearest protein-coding gene identified by using UCSC Genome Browser at a distance of <10 kb.

### LncRNAs are correlated with coding gene expression

To investigate whether lncRNAs might be associated with reduced nephron endowment, correlation analysis between differentially expressed lncRNAs and mRNAs involved in kidney development was performed. Although most of the differentially expressed lncRNAs have not been functionally characterized, we identified 16 lncRNAs correlated with the mRNA expression of mitogen-activated protein kinase 4 (MAPK4) and 6 lncRNAs (TCONS_00116697, TCONS_00031111, ENSRNOT00000004831, TCONS_00115368, TCONS_00049728, and TCONS_00106832) correlated with the expression of both MAPK4 and MAPK10 mRNAs, with most of the pairs presenting a positive correlation. Previous studies have revealed that the MAP kinase pathway is essential for normal branching morphogenesis of the ureteric bud in the developing kidney [26, 27]. These results, indicating that one coding gene could correlate with 16 lncRNAs, implicate the inter-regulation of lncRNAs and mRNAs in the kidneys during nephron development. The correlation analysis between differentially expressed lncRNAs with the mRNA expression of MAPK4 is shown in Table 5.

**Table 5:** The relationship of differentially expressed lncRNAs with the mRNA expression of MAPK4.

LncRNA	r	*P* value
TCONS_00045737	0.97955	0.00062
TCONS_00116697	0.94552	0.00437
TCONS_00106261	0.93735	0.00576
TCONS_00031111	0.93508	0.00619
TCONS_00134054	0.93221	0.00674
TCONS_00031872	0.93182	0.00681
TCONS_00139993	0.93128	0.00692
TCONS_00038464	0.92979	0.00722
ENSRNOT00000004831	0.91042	0.01168
TCONS_00014138	0.90362	0.01349
TCONS_00014139	0.90022	0.01444
TCONS_00049728	-0.98688	0.00026
TCONS_00095270	-0.94912	0.00382
TCONS_00017119	-0.9237	0.00851
TCONS_00115368	-0.90863	0.01214
TCONS_00106832	-0.90277	0.01372

r = Pearson correlation coefficient

MAPK4, mitogen-activated protein kinase 4

### Real-time quantitative PCR validation

**Fig 3:**
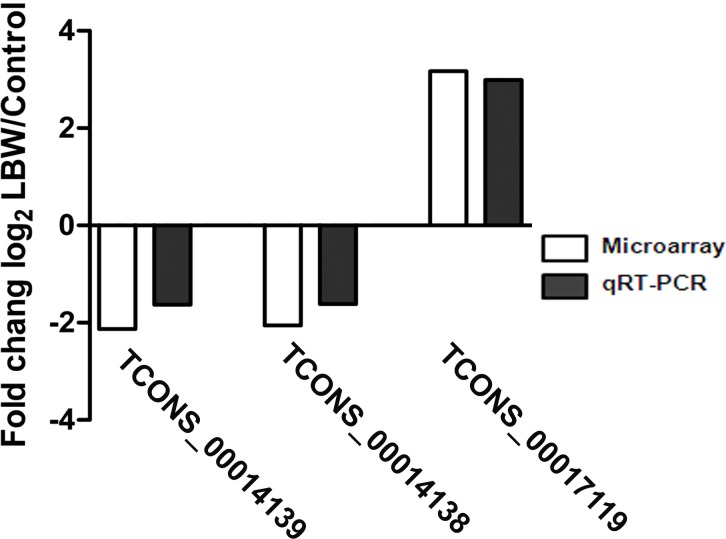
Comparison of microarray and quantitative real-time PCR data for differentially expressed lncRNAs. The qRT-PCR results (n *=* 7) in three lncRNAs are consistent with the microarray data in the kidney obtained at postnatal day 10. The qRT-PCR reactions were repeated three times for every lncRNA. The level of lncRNA was calculated relative to GAPDH. The data are expressed as the log-transformed mean fold changes (Low birth weight/Control).

From the 16 differentially expressed lncRNAs that were significantly correlated with the mRNA expression of MAPK4, we randomly selected 4 of the down-regulated and 1 of the up-regulated lncRNAs with fold change ≥2 (TCONS_00116697, TCONS_00038464, TCONS_0014139, TCONS_00014138, and TCONS_00017119) for qRT-PCR validation. The qRT-PCR results of 3 lncRNAs (TCONS_0014139, TCONS_00014138, and TCONS_00017119) from LBW and control kidneys at p10 (n = 7) were concordant with the microarray data, as shown in Fig 3.

### LncRNAs are differentially expressed in kidneys of LBW rats at postnatal days 1 and 10

**Fig 4:**
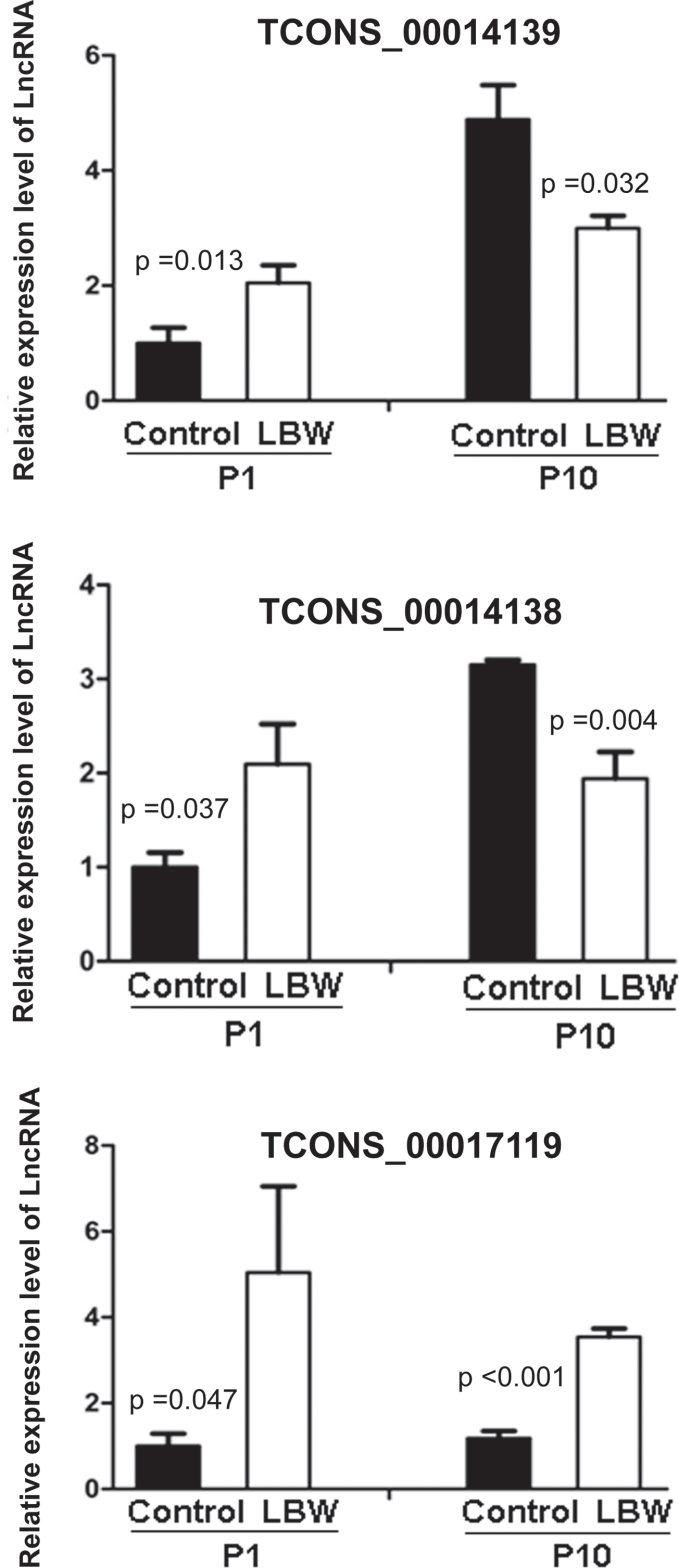
LncRNAs expression in the kidneys of low birth weight rats compared with controls. Comparison of the expression of lncRNAs in kidney between low birth weight (LBW) and control rats at postnatal day 1 (p1) and day 10 (p10) by quantitative real-time PCR. The level of lncRNA was calculated relative to GAPDH. The data are expressed as relative to the controls at p1. Values are means with SEM. *p* values for comparison of lncRNA level between LBW and control rats was calculated by the Student’s t-test (n = 7).

To further investigate whether differentially expressed lncRNAs might be involved in the development of nephron, we analyzed the expression of these three lncRNAs, which were concordant with microarray data and significantly correlated with MAPK4 mRNA, from LBW and normal control kidneys at the two experimental time-points (p1 and p10) by using qRT-PCR. LncRNAs TCONS_0014139 and TCONS_00014138 were down-regulated at p10 after the initial up-regulation at p1, while lncRNA TCONS_00017119 was consistently up-regulated at the two experimental time-points, as shown in Fig 4 (n = 7 for each group at each time point).

### LncRNAs are correlated with glomerular number at postnatal day 10

**Fig 5:**
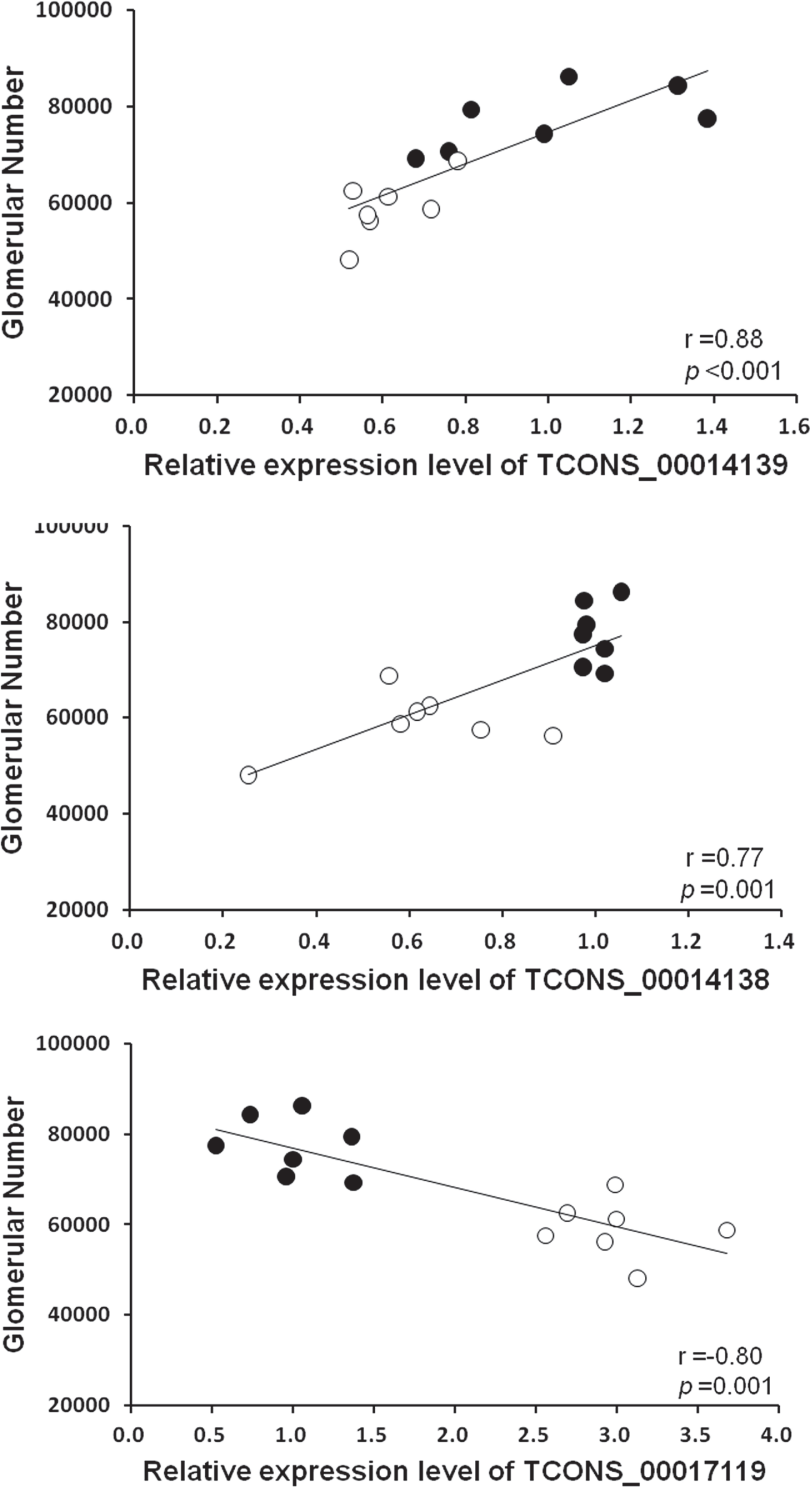
Correlation analysis of lncRNAs expression with glomerular number. The number of glomeruli was positively correlated with the relative expression of lncRNAs TCONS_0014139 (A: r = 0.88; *p* <0.001; n = 14) and TCONS_00014138 (B: r = 0.77; *p* = 0.001; n = 14), but negatively with lncRNA TCONS_00017119 (C: r = -0.80; *p* = 0.001; n = 14) in the kidneys at postnatal day 10 (p10). Black circles, control rats (n = 7); open circles, low birth weight rats (n = 7). The level of lncRNA was calculated relative to GAPDH. The data are expressed as relative to the controls at p10. r = Spearman’s correlation coefficient.

The correlations between these three lncRNAs and glomerular number are shown in Fig 5. The total number of glomeruli per kidney was significantly positively correlated with the relative expression level of lncRNAs TCONS_0014139 and TCONS_00014138, but negatively with lncRNA TCONS_00017119, at p10.

### LncRNAs expression patterns in different tissues at postnatal day 10

**Fig 6:**
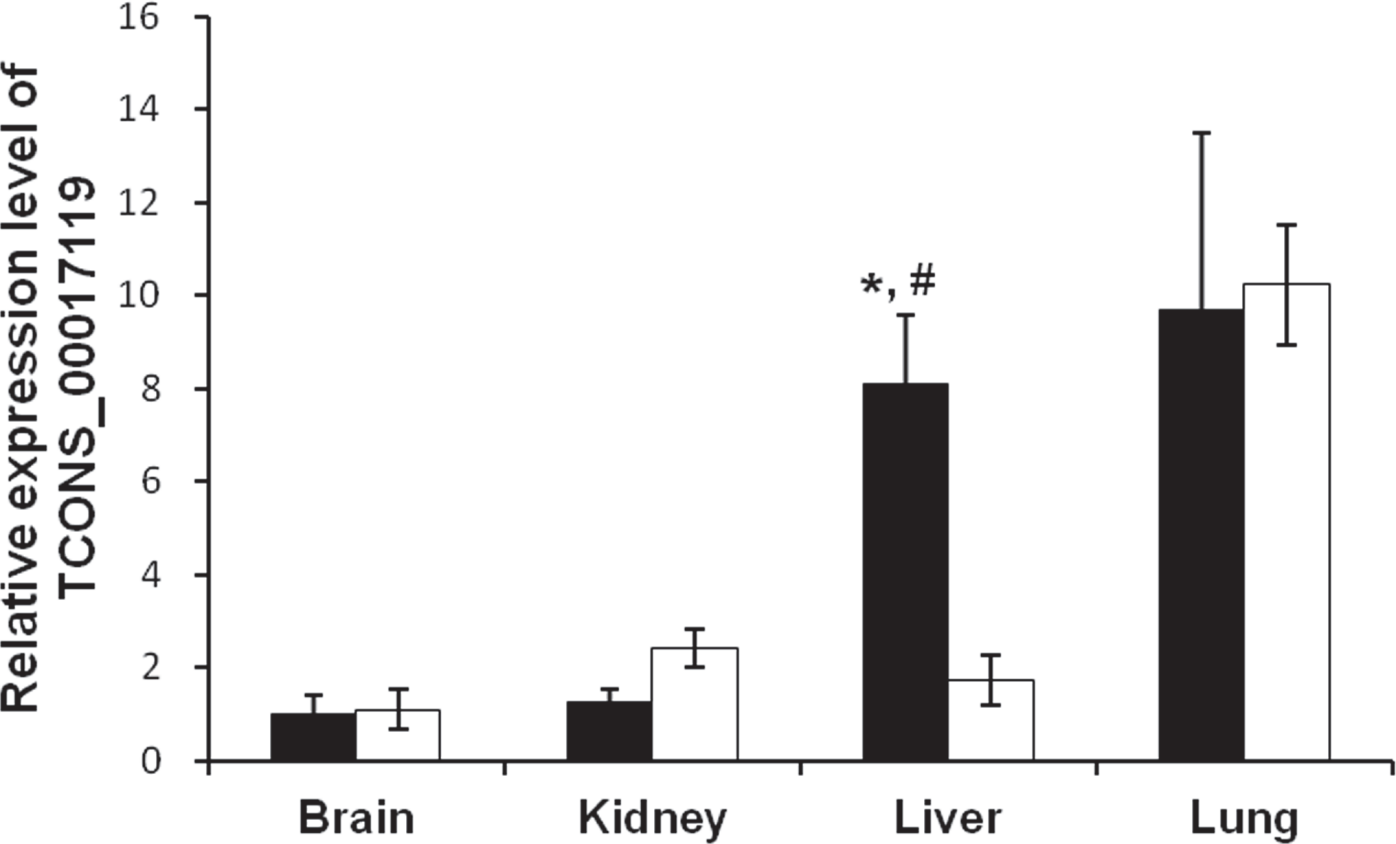
Comparison of lncRNA expression in different tissues. Comparison of the expression of lncRNA TCONS_00017119 in different tissues from low birth weight and control rats at postnatal day 10 by quantitative real-time PCR. The level of lncRNA was calculated relative to GAPDH. The data are expressed as relative to lncRNA level in the brain of normal control rats. Values are means with SEM. *p* Value was calculated by the Student’s t-test. Black bars, control rats (n = 5); open bars, low birth weight rats (n = 5). *p <0.05 vs. low birth weight rats, ^#^p <0.05 vs. normal kidney.

To determine whether the differential expression of lncRNAs was specific to the kidneys, we examined the expression level of these three lncRNAs in kidney, liver, brain, and lung tissues, in a separate experiment, from LBW and normal control rats at p10 using qRT-PCR (n = 5 for each group). The expression of lncRNA TCONS_0014139 was negligible in the rat tissues of liver, brain, and lung, suggesting that TCONS_0014139 expression might be specific to the kidneys. LncRNA TCONS_00014138 was detectable in normal liver and lung. However, the expression of TCONS_00014138 in LBW rat tissues of liver (*p* = 0.987) and lung (*p* = 0.212) did not significantly differ from that in controls at p10. LncRNA TCONS_00017119 was detected in rat tissues of liver, brain, and lung, and expressed at high levels in normal liver and lung. As shown in Fig 6, the expression of TCONS_00017119 was higher in normal liver than in kidney (*p* = 0.010). There was a significant decrease in the expression of TCONS_00017119 in liver of LBW rats compared with normal controls at p10 (*p* = 0.009). There was no significant difference in the expression of TCONS_00017119 in normal lung compared with normal kidney (*p* = 0.092). There was also no difference in TCONS_00017119 expression in lung of LBW rats compared with controls (*p* = 0.896). These results indicate that the changes of these potential lncRNAs expression in LBW rats might be specific to the kidneys.

## Discussion

In this study, we have characterized the expression profile of lncRNAs in LBW rat kidneys with reduced nephron endowment, which was induced by maternal protein restriction, using microarray analysis. After confirmation of microarray by qRT-PCR, significant differences in lncRNA expression profiles were observed in the kidney at two time-points of nephron development (postnatal days 1 and 10), thus suggesting that lncRNAs might be involved in reduced nephron endowment.

LncRNAs are generally considered to be expressed at lower levels, compared to protein-coding genes and to be more likely to display a tissue-specific pattern of expression [15, 28, 29]. To identify the significantly up/down-regulated lncRNAs in the kidneys during nephron development, the differential expression of lncRNAs between LBW and control rats was assessed using a microarray with abundant and varied probes accounting for both lncRNAs and coding transcripts. Based on our microarray data, 8,941 lncRNAs were detected above the background in LBW rat kidneys at postnatal day 10, and most of them have not been functionally characterized. Notably, we identified total of 42 lncRNAs that were significantly differentially expressed in LBW rats compared with normal controls, including 25 lncRNAs that were down-regulated and 17 lncRNAs that were up-regulated.

LncRNAs are required for the development of organ, such as the heart and brain [17, 18]. In renal development in rats, the formation of nephrons continues after birth until postnatal days 7–10 [21]. In this study, LBW rats exposed to maternal protein restriction during postnatal nephron development had significantly reduced body weight and nephron endowment. Our findings might suggest that lncRNAs might be involved in reduced nephron endowment. In accordance with our results, it was previously described that lncRNA H19 is developmentally regulated in the kidney and might be responsible for epithelial and mesenchymal interactions [20]. Nephrogenesis, the formation of nephrons, commences when the ureteric bud invades the metanephric mesenchyme, causing the ureteric bud to grow and undergo branching [21].

Among a number of randomly selected differentially expressed lncRNAs validated by qRT-PCR, 3 lncRNAs (TCONS_00014139, TCONS_00014138, and TCONS_00017119), which were concordant with the microarray data, were selected for comparison analysis between LBW and control kidneys at the two time-points of postnatal days 1 and 10 using qRT-PCR. The dynamic change of these potential lncRNAs expression during postnatal nephron development and the significant correlation between the differentially expressed lncRNAs and glomerular number may provide evidence that lncRNAs might play significant biological roles in reduced nephron endowment.

Although the biological functions of these three lncRNAs remain unclear, they have a close relationship with the mRNA expression of MAPK4. The protein encoded by the mRNA is a member of the MAP kinase family. This kinase pathway is essential for the normal branching morphogenesis of the ureteric bud in the developing kidney [26, 27]. In this study, the qRT-PCR results demonstrated that these three lncRNAs were aberrantly expressed in LBW kidneys at both postnatal day 1 and 10. We propose that these three lncRNAs might play a role in reduced nephron endowment by interacting with MAPKs or contributing to the regulation of other genes. Further studies are required to identify the functional role of these potential lncRNAs in nephron development and to determine how these lncRNAs are involved in the regulation of related genes.

## Conclusions

This is the first study to examine the expression profiles of lncRNAs in LBW rats during postnatal nephron development. A number of lncRNAs were aberrantly expressed at two time-points of nephron development in the kidneys of LBW rats with reduced nephron endowment compared with normal controls. Our data indicate that these lncRNAs might play a potential role in impaired nephron endowment and provide a basis for future investigations on nephron development.

## Supporting Information

S1 Table LncRNAs differentially expressed in the kidneys of low birth weight rats compared with controls at postnatal day 10. (XLS)

S2 Table mRNAs differentially expressed in the kidneys of low birth weight rats compared with controls at postnatal day 10. (XLSX)
